# The “Old yet New” Echocardiographic Finding for Constrictive Pericarditis Following Purulent Pericarditis: A Case Report

**DOI:** 10.7759/cureus.64057

**Published:** 2024-07-08

**Authors:** Yuta Sudo

**Affiliations:** 1 Cardiology, Soka Municipal Hospital, Saitama, JPN

**Keywords:** colchicine therapy, streptococcus pyogenes infection, echocardiography, constrictive pericarditis, purulent pericarditis

## Abstract

Purulent pericarditis (PP) is a localized infection of the pericardial cavity with suppuration that can be life-threatening. Treatment for PP consists of pericardial drainage and antimicrobial therapy. Constrictive pericarditis (CP), a form of diastolic heart failure that arises because an inelastic thickened pericardium, is a possible related dreadful complication of PP. Several echocardiographic findings suggestive of CP have been reported, but some require measurements or are difficult to reproduce. This case report presents a simple echocardiographic finding that reflects the clinical course of transient CP (TCP). A 76-year-old Japanese man presented to our hospital with chest pain and dyspnea. He was diagnosed with PP caused by *Streptococcus pyogenes* and treated with pericardial drainage and benzylpenicillin. The response to the treatment of the infection was favorable, but subsequent echocardiography and cardiac catheterization revealed a CP complication. Treatment with colchicine and ibuprofen was initiated, with improvement in CP within three months. During CP, a restricted right ventricular (RV) motion and movement of the liver towards the heart were observed before other echocardiographic findings suggestive of CP. Furthermore, this echocardiographic finding disappeared and normalized as CP improved. In this case of TCP following PP, changes in the echocardiographic “RV sliding” sensitively reflected the clinical course of CP. This simple finding may indicate inflammation of the pericardium and could be useful for the diagnosis and follow-up of CP.

## Introduction

Purulent pericarditis (PP) is a rare, life-threatening localized infection of the pericardial space with a mortality rate of 20%-30%, which can be treated by pericardial drainage and antimicrobial therapy [1]. Constrictive pericarditis (CP) is a clinical syndrome where an inelastic thickened pericardium restricts cardiac filling, and it can occur during the course of PP [2]. A subset of CP improves spontaneously or with medical therapy and is referred to as transient CP (TCP). Several echocardiographic findings characteristic of CP have been reported [3,4]. Here, I report a case of PP-associated TCP that presented with other simple echocardiographic findings reflecting the clinical course of the disease.

## Case presentation

**Figure 1:**
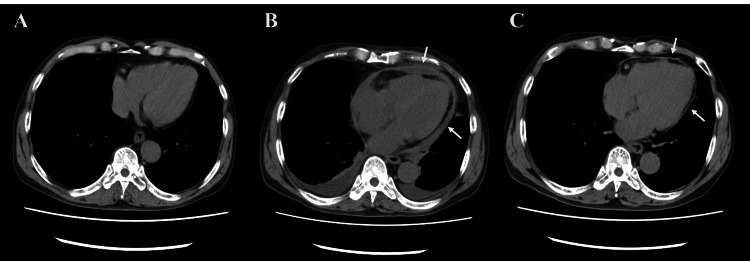
Sequential changes in chest computed tomography. (A) Two years before admission (control). (B) Day 18. Three-layered pericardium (arrow). (C) Day 119. Improved pericardial thickening (arrow).

A 76-year-old Japanese man presented with excruciating chest pain, palpitations, and dyspnea persisting for 10 days with no apparent change in breathing or body positioning symptoms. Besides antihypertensive medication for the past 10 years, the patient had no significant medical history, including chest surgery, endoscopic examinations, chemotherapy, smoking, heavy alcohol consumption, drug abuse, overseas travel, or pet ownership. Two years earlier, computed tomography (CT) performed during a health check-up detected no pericardial effusion or thickening (Figure 1A).

**Figure 2:**
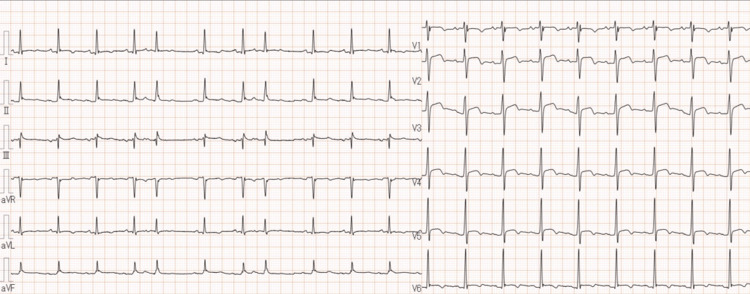
Electrocardiogram on admission. Diffuse ST elevation and PR depression were observed.

At admission, his blood pressure was 129/91 mmHg, heart rate 95 beats/min, body temperature 37.2 °C, and oxygen saturation 95% with 1 L/min oxygen supplementation. No pericardial friction rubbing, decreased breathing sounds, or pulsus paradoxus were observed. Electrocardiography revealed diffuse changes in the ST and PR segments (Figure 2). Blood tests revealed an elevated white blood cell (WBC) count (19,900/μL; normal range 3,300-9,000/μL), neutrophilia, and high levels of C-reactive protein (CRP) (36.58 mg/dL; normal range <0.30 mg/dL), presepsin (1020 pg/mL; normal range <500 pg/mL), and brain natriuretic peptide (BNP) (181.6 pg/mL; normal range <18.4 pg/mL) (Table 1).

**Table 1:** Blood test results at admission WBC: white blood cell, CRP: C-reactive protein, BNP: brain natriuretic peptide

Items	Result	Reference range
WBC	19,900/μL	3,300-9,000/μL
CRP	36.58 mg/dL	< 0.30 mg/dL
Presepsin	1,020 pg/mL	< 500 pg/mL
BNP	181.6 pg/mL	< 18.4 pg/mL

**Figure 3:**
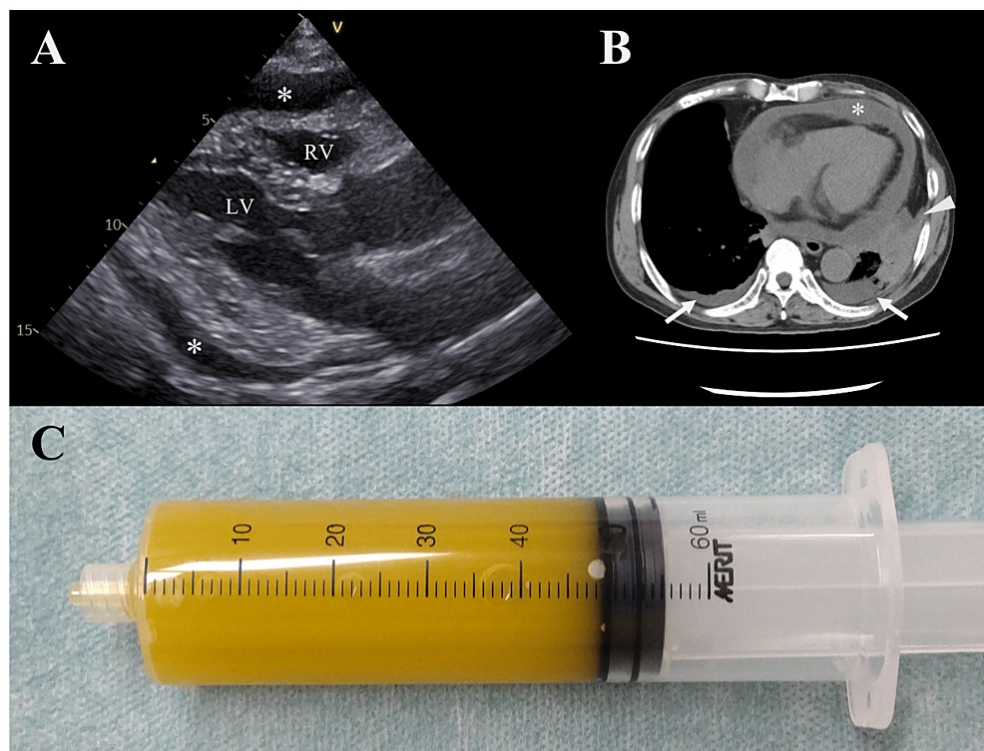
Purulent pericardial effusion. (A) Pericardial effusion on the parasternal long-axis view on echocardiography (asterisk). (B) Pleural effusion observed on chest computed tomography (arrow), atelectasis (arrowhead), and circumferential pericardial effusion (asterisk). (C) Yellowish turbid pericardial fluid collected by pericardiocentesis.

Transthoracic echocardiography revealed moderate pericardial effusion (Figure 3A) without cardiac tamponade while transesophageal echocardiography revealed no obvious vegetations. Chest and abdominal CT showed circumferential pericardial effusion, left-dominant pleural effusion, and left lower lobe atelectasis (Figure 3B), without any signs of pneumonia, lung abscess, or abdominal organ infection. Furthermore, two sets of pre-antibiotic administration blood cultures were negative. Thoracentesis revealed the presence of lymphocytes and neutrophils in the culture-negative yellow transparent pleural fluid; however, the yellowish turbid pericardial fluid (500 mL), drained by pericardiocentesis, had a significantly high cell count (48,853/μL, 97.5% segmented neutrophils) (Figure 3C). Over the next five days, 1,000 mL of pericardial fluid was drained, and its analysis led to the diagnosis of PP with *Streptococcus pyogenes* infection. The pericardial fluid culture was negative for *Mycobacterium tuberculosis*, fungi, or other pathogens; and the patient did not have any malignancy. Ceftriaxone (2 g/24 h) and vancomycin (25 mg/kg initial dose) were administered post-pericardiocentesis, which was replaced with benzylpenicillin (24 million units/day) upon confirmation of the microbiological tests on day 8. Blood inflammatory marker levels declined significantly from day 2, with WBC and presepsin levels returning to normal shortly thereafter, while CRP remained at approximately 5 mg/dL around day 18 with no further improvement. Antibiotic therapy was discontinued from day 25.

**Figure 4:**
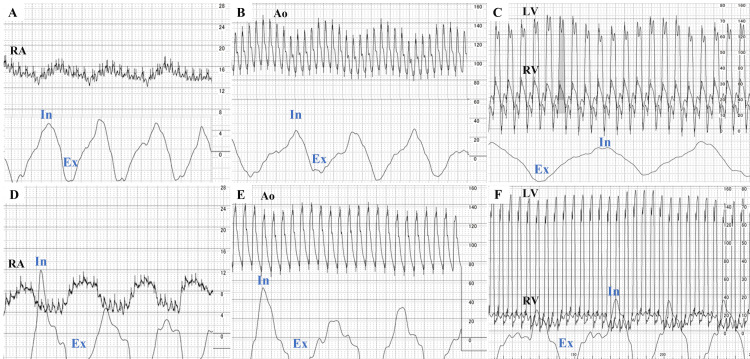
Cardiac catheterization. (A-C) Day 25. (A) Kussmaul's sign, (B) pulsus paradoxus, (C) RV-LV pressure discordance with respiratory cycle. (D-F) Day 119. Ao = Aorta, LV = left ventricle, RV = right ventricle, Ex = expiration, In = inspiration.

However, from approximately day 17, the heart rate gradually increased while maintaining sinus rhythm. On day 18, a chest CT showed pericardial thickening (Figure 1B), while an echocardiogram revealed a septal bounce, increased mitral medial e', and dilated inferior vena cava (IVC) (Table 2). In the subxiphoid view, smooth motion of the right ventricular (RV) free wall in the longitudinal direction was restricted, and the liver was being pulled towards the heart during the systole (Video 1). This finding was first observed on day 8. On day 25, a catheter examination revealed a clear Kussmaul’s sign, pulsus paradoxus, and respiratory ventricular systolic discordance (Figures 4A-4C). In conjunction with other test results (Table 3), the patient was diagnosed with PP-associated CP. Treatment with colchicine (0.5 mg) and ibuprofen (600 mg) once and thrice per day, respectively, was initiated; the patient was discharged on day 26.

**Table 2:** Echocardiographic data timeline ^a^units are in beats/min; ^b^units are in cm/s; ^c^after pericardiocentesis E/A: mitral valve E velocity divided by A-wave velocity, HR: heart rate, IVC: inferior vena cava, RV: right ventricle

Day	HR^a^	E/A	Mitral medial e^b^	IVC dilation	Septal bounce	RV sliding
1^c^	76	0.92	5.5	-	-	+
4	83	0.66	6.5	-	-	+
8	90	0.92	6.7	-	-	-
11	88	1.31	6.7	-	-	-
18	101	1.07	9.3	+	+	-
24	107	1.08	10.3	+	+	-
42	98	1.86	8.1	+	+	-
66	101	0.59	5.1	+	-	+
119	73	0.6	4.9	-	-	+
154	63	0.6	6.4	-	-	+

**Video 1:** Subxiphoid view in echocardiography (day 18).

**Table 3:** Catheterization data (days 25 and 137) d: diastolic, e: end-diastolic, m: mean, s: systolic. On day 25, the superior vena cava and right atrial pressures were elevated. The right ventricular end-diastolic pressure increased to approximately the same level as the left ventricular one. On day 137, all findings improved.

Parameters	Day 25	Day 137
Superior vena cava (mmHg)	18	8
Right atrium (mmHg)	18	8
Kussmaul's sign	+	-
Right ventricle (s/d/e) (mmHg)	29/13/19	27/8/12
Pulmonary artery (s/d/m) (mmHg)	23/18/20	24/13/18
Pulmonary Artery Wedge (m) (mmHg)	16	11
Aorta (s/d/m) (mmHg)	137/93/111	134/73/100
Pulsus paradoxus	+	-
Left ventricle (s/d/e) (mmHg)	140/1/20	140/1/18
Cardiac output (L/min)	3.8	6.1
Cardiac index (L/min/m^2^)	2.3	3.7
Heart rate (/min)	102	71

On day 42, the patient complained of mild dyspnea and bilateral leg oedema. Furosemide (20 mg) was administered after chest radiography, which showed significant increase in the pleural effusion; echocardiography findings were similar to those taken pre-discharge. On day 66, his dyspnea improved, and pleural effusion was observed to be significantly decreased on chest radiography. While the septal bounce and abnormal findings in the subxiphoid view almost disappeared on echocardiogram (Video 2), his sinus tachycardia, leg edema, elevated BNP (180.7 pg/mL), and IVC dilatation persisted. Since his symptoms improved on day 119, administration of colchicine, ibuprofen, and furosemide was discontinued. Eighteen days after discontinuation, a heart catheter examination showed CP improvement (Figures 4D-4F, Table 3). A CT scan showed improved pericardial thickening (Figure 1C). During follow-up on day 154, the patient did not complain of any specific symptoms, and there were no abnormal findings in the subxiphoid view on echocardiography (Video 3).

**Video 2:** Subxiphoid view in echocardiography (day 66).

**Video 3:** Subxiphoid view in echocardiography (day 154).

## Discussion

I have presented a case of PP-associated CP, which was successfully treated with conservative anti-inflammatory therapy alone. As causative agents of PP, *Staphylococcus aureus*, *Streptococcus pneumoniae*, and fungi are common [5]. PP caused by *S. pyogenes* is rarely reported, mostly involving infants and children, making this patient the oldest among previously reported cases (Table 4). Additionally, while cardiac tamponade has been frequently reported as a complication of PP caused by this bacterium, this case is the first to document the development of CP. Although the mechanisms for developing PP include direct extension from pneumonia, hematogenous spread, and infective endocarditis, this case showed none of these associations, leaving the mechanism unknown [5].

**Table 4:** Purulent Pericarditis Caused by Streptococcus pyogenes AR: aortic regurgitation, VF: ventricular fibrillation, M: male, F: female

Reference	Age	Sex	Complication
Vigneswaran WT, et al. [6]	14 years	M	cardiac tamponade
Pruitt JL. [7]	2 years	F	death
Thébaud B, et al. [8]	13 months	M	cardiac tamponade
Thébaud B, et al. [8]	3 years	F	cardiac tamponade
Thébaud B, et al. [8]	14 months	F	cardiac tamponade
Barth H, et al. [9]	16 months	F	cardiac tamponade, mycotic pseudoaneurysm
Bhaduri-McIntosh S, et al. [10]	6 years	M	cardiac tamponade
Megged O, et al. [11]	3 years	F	cardiac tamponade
Angoulvant F, et al. [12]	3 years	F	cardiac tamponade
Schwartz MC, et al. [13]	10 months	M	cardiac tamponade
Pemira SM, et al. [14]	4 years	M	cardiac tamponade, splenic abscesses
Yamasaki M, et al. [15]	20 years	M	commissural avulsion, AR
Giudicatti LC, et al. [16]	64 years	M	cardiac tamponade
Al-Waili BR, et al. [17]	4 months	F	cardiac tamponade
Fry E, et al. [18]	18 years	F	cardiac tamponade, VF, mycotic pseudoaneurysm
Chung N, et al. [19]	2 years	M	none
Higuchi T, et al. [20]	10 years	F	infectious aneurysms
Tarun S, et al. [21]	4 years	F	pericardial mass
This report	76 years	M	constrictive pericarditis

The current case suggests that the combination of restricted motion of the RV free wall and the movement of the liver due to the adhesion between the parietal pericardium and the liver, which causes the liver to be pulled towards the heart with each heartbeat, in the subxiphoid view on echocardiography may be useful for the diagnosis and follow-up of CP after PP. Suggestive signs of CP on echocardiography include a mitral inflow pattern and a ventricular septal motion abnormality [22], but these can be difficult to assess because of tachycardia or lack of operator skill. The findings observed in the subxiphoid view in this case were earlier considered as signs of CP complicated by open-heart surgery [23]. In the present case, this “RV sliding” not only disappeared during the phase of complication of CP but also reappeared when CP improved (Table 1), suggesting that this “old yet new” finding may reflect inflammation of the pericardium. The absence of RV sliding manifested earlier than other characteristic echocardiographic findings, suggesting that this finding in patients with PP may lead to early additional tests, diagnosis, and treatment of CP. In addition, this sign may have the advantage of being much simpler, quicker, and more reproducible than the previously useful findings.

The limitation of this case report is that cardiac magnetic resonance imaging could not be performed when CP was suspected. Consequently, imaging evidence of inflammation in the pericardium could not be provided [24]. However, the echocardiographic finding of RV sliding may serve as a simpler and more cost-effective supplementary sign.

## Conclusions

In conclusion, *S. pyogenes* can cause PP and subsequent CP in the elderly. The echocardiographic assessment of “RV sliding” may be useful and feasible for the diagnosis and follow-up of PP-associated CP. Whether this finding will be useful for other patients with CP remains to be seen and awaits future reports.
